# Cellular changes in boric acid-treated DU-145 prostate cancer cells

**DOI:** 10.1038/sj.bjc.6603009

**Published:** 2006-02-21

**Authors:** W T Barranco, C D Eckhert

**Affiliations:** 1 Department of Environmental Health Sciences, University of California, Los Angeles, Box 951770, CA 90095-1772, USA

**Keywords:** boric acid, prostate cancer, DU-145, migration, senescence, acidosis

## Abstract

Epidemiological, animal, and cell culture studies have identified boron as a chemopreventative agent in prostate cancer. The present objective was to identify boron-induced changes in the DU-145 human prostate cancer cell line. We show that prolonged exposure to pharmacologically-relevant levels of boric acid, the naturally occurring form of boron circulating in human plasma, induces the following morphological changes in cells: increases in granularity and intracellular vesicle content, enhanced cell spreading and decreased cell volume. Documented increases in *β*-galactosidase activity suggest that boric acid induces conversion to a senescent-like cellular phenotype. Boric acid also causes a dose-dependent reduction in cyclins A–E, as well as MAPK proteins, suggesting their contribution to proliferative inhibition. Furthermore, treated cells display reduced adhesion, migration and invasion potential, along with F-actin changes indicative of reduced metastatic potential. Finally, the observation of media acidosis in treated cells correlated with an accumulation of lysosome-associated membrane protein type 2 (LAMP-2)-negative acidic compartments. The challenge of future studies will be to identify the underlying mechanism responsible for the observed cellular responses to this natural blood constituent.

The element boron is nearly completely absorbed from drinking water and plant-derived foods in the gastrointestinal tract, and circulates in blood as boric acid (BA) (Price *et al*, 1997). Cells were once thought incapable of processing the element, yet this has since been disproved. Boron is utilised by bacteria in the structure of several antibiotics and autoinducer-2, a signalling molecule utilised during interspecies quorum sensing (Chen *et al*, 2002; Semmelhack *et al*, 2004). Plants require the element for growth, flowering and seed formation, and obtain boron from soil pore water using a borate transporter, BOR1, expressed in root pericycle cells (Takano *et al*, 2002). A human homologue, the electrogenic, voltage-regulated, Na^+^-coupled borate transporter NaBC1, was recently identified in human kidney tubular cells and may function to maintain plasma BA levels (Park *et al*, 2004).

There are several reports supporting boron as a chemopreventative agent against prostate cancer. An epidemiological study using data from the NHANES III database reported that the risk of prostate cancer in US men is inversely proportional to dietary intake of boron (Cui *et al*, 2004). The biological plausibility of this observation has been supported by cell culture and animal studies. Treatment of nude mice, injected with androgen-sensitive LNCaP prostate cancer cells, with BA caused a reduction in tumour growth of 25–38%, along with a reduction in plasma PSA levels of 88% (Gallardo-Williams *et al*, 2004). BA inhibits the activity of serine proteases, including prostate-specific antigen (PSA), presumably by binding to its active site (Bone *et al*, 1987; Gallardo-Williams *et al*, 2003). In culture, BA has been shown to inhibit the proliferation of LNCaP and the androgen-independent prostate cancer cell lines DU-145 and PC-3, in a dose-dependent manner (Barranco and Eckhert, 2004). Since DU-145 cells do not synthesise PSA, BA's mode of inhibiting proliferation is likely not to occur by inhibiting the conversion of IGFBP-3 to IGF-1, as proposed in LNCaP tumours (Gallardo-Williams *et al*, 2004; Sobel and Sadar, 2005). The present investigation was initiated to define morphological and molecular responses of DU-145 prostate cancer cells to BA, which might lead to an explanation of its antiproliferative properties.

In the current report, we examined the effects of pharmacological concentrations of BA on cell morphology and molecular markers of proliferation, senescence, metastasis and motility. We show that prolonged exposure to BA causes DU-145 cells to develop a flattened, angular phenotype with numerous vesicles appearing in the cytoplasm. These changes occur coincident with a decrease in the expression of cyclin proteins, p21 and P-MEK1/2, as well as a reduction in cell motility and invasion capacity. Finally, increased *β*-galactosidase activity reflects a conversion of DU-145s to a senescence-like cell.

## MATERIALS AND METHODS

### Experimental culture

DU-145, LNCaP, and PC-3 PCa cells, donated by Dr Allan Pantuck, were cultured in RPMI 1640 media (Invitrogen, USA) supplemented with 10% FBS, penicillin/streptomysin (100 U ml^−1^; 100 *μ*g ml^−1^), and L-glutamine (200 mM) (Gemini Bioproducts, USA). Experimental media was prepared as previously published in Barranco and Eckhert (2004). Cells were plated directly onto culture plates or glass coverslips and allowed to settle overnight in nontreated media. After 24 h, media was aspirated and replaced daily, for 7–8 days, with BA-supplemented media (0–1000 *μ*M). Cell counts were performed using a hemacytometer and Trypan Blue (Invitrogen) for identifying nonviable cells.

### Flow cytometry

Following 8 days in culture with BA (0, 250, and 1000 *μ*M), DU-145 cells were trypsinised, resuspended as 1 ml aliquots (10^6^ cells ml^−1^) in loading buffer (RPMI 1640 w/o phenol red), and incubated in 12 × 75 mm polystyrene test tubes for 30 min, at 37°C, 5% CO_2_. Following incubation, forward light scatter and side light scatter analysis (serving as measures of cell size and granularity, respectively) were determined using a Becton Dickinson BD-LSR analytic flow cytometer on samples of 10 000 cells. Data analysis was performed with FLOWJO. Loading buffer was supplemented with Indo-1 AM (1 *μ*M) (Sigma, USA), a cell-permeable Ca^2+^ fluorescent probe, for concordant measurements of intracellular calcium.

### *β*-galactosidase assay

Detection of *β*-galactosidase activity was determined using a previously published procedure (Dimri *et al*, 1995). DU-145 cells (8-day exposed), cultured on glass coverslips, were washed in PBS and fixed in 3% formaldehyde for 5 min. Fixed cells were washed with PBS and incubated overnight at 37°C (low CO_2_) with fresh *β*-Gal stain solution (1 mg ml^−1^ 5-bromo-4-chloro-3-indoyl *β*-D-galactopyranoside (X-Gal), 40 mM citric acid/40 mM sodium phosphate (pH 4.0 & 6.0), 5 mM potassium ferrocyanide, 5 mM potassium ferricyanide, 150 mM NaCl, 2 mM MgCl_2_). The percentage of cells testing positive for *β*-galactosidase activity (appearing blue) in four randomly selected optical fields were determined under light microscopy.

### Western blot

DU-145 cells were cultured for 1, 2, and 7 days in the presence of BA (0–1000 *μ*M), on 100 × 20 mm tissue culture plates, with daily media replacement. Following treatment, monolayers were washed with PBS, removed with a rubber policeman, and centrifuged at 1200 rpm for 5 min. For protein extraction, pellets were submerged in lysis buffer (250 mM NaCl, 0.1% NP40, 50 mM HEPES (pH 7.0), 5 mM EDTA, 1 mM DTT, 10% protease inhibitor mixture (Sigma)), sonicated, and incubated for 40 min at 4°C. Wells of 10% stacking, 12% separating (SDS–PAGE) gels were loaded with 30 *μ*g of protein per sample and separated for 30 min at 100 V, followed by 1 h at 200 V. Separated proteins were transferred to nitrocellulose membranes for 4 h at 40 V, 4°C. Membranes were blocked overnight (Nonfat dry milk 4 g, 38 mM Tris base, 125 mM NaCl 2.5, 100 *μ*l Tween 20, ddH_2_O 100 ml). The 2-h primary antibody exposure (1/200–1/800 dilution) was followed by a 10 min wash in PBS/Tween 20 (0.1%) and 1-h secondary antibody exposure (1/1000 dilution) was followed by 3 × 10 min washes in PBS/Tween 20 (0.1%). Probed membranes were submerged in ECL detection reagent, (Amersham, USA), wrapped in cellophane, and exposed to X-ray film (Fuji). All primary and secondary antibodies were purchased from Santa Cruz Biotechnology (Santa Cruz, CA, USA).

### Fluorescent probe detection of actin and acidic compartments

For actin probing (F-actin, fluorescein phalloidin; G-actin, fluorescent deoxyribonuclease I conjugate) (Molecular Probes, USA), 8-day BA-treated DU-145 cells were washed 2 × with PBS and fixed in PBS containing 3.7% formaldehyde, for 10 min at room temperature. Fixed cells were washed 2 × with PBS before being extracted with acetone (−20°C) for 5 min. 2 × wash with PBS followed before cells were loaded with phalloidin (0.16 *μ*M in 1% BSA/PBS) or deoxyribonuclease I (0.3 *μ*M in glycerol/PBS) for 20 min, at 37°C. Loaded cells were washed 2 × with PBS, mounted on slides, and viewed under confocal microscopy (Fluorescein: ex 496, em 516).

For intracellular acidic compartment labelling, 8-day BA-exposed DU-145 cells were loaded with a nonspecific lysosome marker (LysoTracker Green) (Molecular Probes). Cells were submerged in prewarmed media containing LysoTracker (1 *μ*M) for 1 h, at 37°C. Following incubation, loading medium was aspirated, replaced with PBS, and cells were viewed under confocal microscopy (ex 504, em 511). All fluorescent images, along with light images, were recorded using an Axioskop 2 FS confocal microscope and brightened using Photoshop 6.0.

### Cell attachment, migration, and invasion assays

For cell attachment efficiency calculations, DU-145 cells were cultured in the presence of BA (0, 250, and 1000 *μ*M) for 8 days on 100 × 20 mm tissue culture plates, trypsinised and replated onto six-well polystyrene culture plates (Fisher, USA) at 2.5 × 10^5^ cells well^−1^. Following a 24-h incubation, nonadherent cells and media were aspirated, while attached cells were trypsinised and counted.

The migration analysis protocol was identical to the attachment assay's, except that 2.5 × 10^5^ cells were loaded in the upper migration chamber of a Corning transwell permeable support (24-well transwell, 8-*μ*M polycarbonate membrane) in 0.1 ml of serum-free RPMI-1640 media. RPMI-1640 (0.6 ml) supplemented with 10% FBS, serving as a chemo-attractant, was deposited in the lower chamber. Plates were covered and incubated for 24 h at 37°C, 5% CO_2_. Following incubation, cells remaining on the upper filter were removed with a cotton swab, while the migrated population on the filter underside was washed with PBS, fixed in methanol, stained with Giemsa stain, rinsed with PBS, and deionised water, and allowed to air-dry. Cells in four random optical fields were counted to determine the number of migratory cells.

The invasion assay procedure was identical to that used in the migration analysis, except that each filter, prior to loading, was coated with 20 *μ*g of growth factor reduced-matrigel (BD biosciences) in 100 *μ*l of cold, serum-free RPMI-1640 media, and subsequently allowed to air-dry overnight in a sterile culture hood.

Since plating efficiency varied among BA-treated cells on matrigel-treated and untreated polycarbonate membranes, test cells were cultured alongside experimental cells and after incubation were trypsinised from filters, counted on a haemacytometer and used to determine the motility fraction.

### Media pH measurements

Following 8-day BA (250 and 1000 *μ*M) exposures to DU-145 cells, with daily media refreshment, media used for the 24 h period between day 7 and 8 was removed and the pH was measured using a Pinnacle 530 pH meter (Corning). Cell counts were performed on the adherent cells from corresponding plates and utilised for calculating the pH shift per cell:

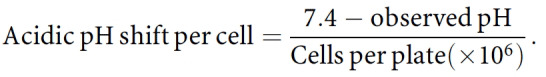


### Statistics

SigmaStat 3.1 statistical software (Systat Software, Point Richmond, CA, USA) was utilised for paired *t*-test. All experiments were performed in triplicate.

## RESULTS

### BA alters cell morphology, while inducing cellular senescence

**Figure 1:**
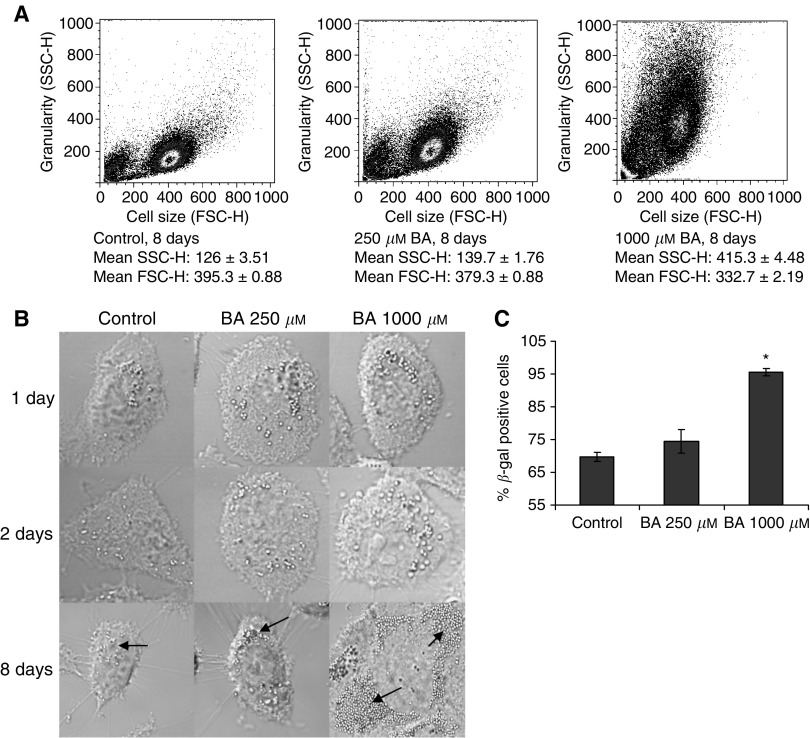
BA-induced morphological and senescent effects, following 8-day exposure, in DU-145 cells. (**A**) Dose-dependent increase in granularity, by way of forward light scatter (FSC-H), and decrease in cell size, by way of side light scatter (SSC-H), from BA (0–1000 *μ*M) exposure, as presented on density plot; mean±s.e.m., *n*=3. (**B**) Confocal images (63 ×) showing dose-dependent intracellular vesicle accumulation, as indicated by arrows, and flattened appearance at 8 days of exposure; whereas at 1 and 2 days cells remain unaltered. (**C**) Dose-dependent *β*-galactosidase activity (pH 4.0) increase in DU-145 cells; mean±s.e.m., *n*=4. ^*^ Statistically significant from control exposures (*P*-value <0.001).

Flow cytometry and light microscopy were used to assess morphological alterations resulting from BA exposure. Following an 8-day exposure to BA (0, 250, and 1000 *μ*M), flow cytometry analysis showed a dose-dependent increase in cellular granularity (side light scatter) and a decrease in cell size (forward light scatter) (Figure 1A). No differences in cell morphology were apparent between confocal images of treated and untreated cells during the first 2 days. By day 8, treated DU-145 cells became flattened and contained numerous vesicles (Figure 1B).

BA's ability to inhibit cell proliferation without cell-death inspired our investigation to determine its effects on markers of senescence. The activity of *β*-galactosidase at pH 4.0, a marker of senescence, increased with BA exposure in a dose-dependent manner (Figure 1C). Enzyme activity was not detected at pH 6.0 (data not shown).

### BA alters proliferation-relevant protein expression

**Figure 2:**
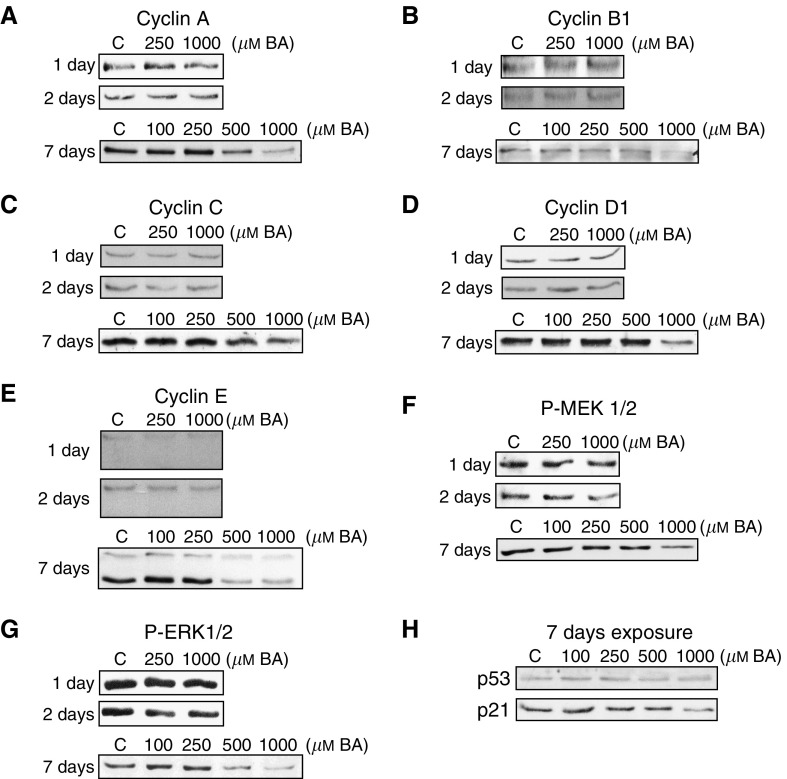
BA-induced alterations in proliferation-relevant protein expression, following 1, 2, and 7-day exposure, in DU-145 cells. (**A**–**E**) Western blots show dose-dependent BA-induced (0–1000 *μ*M) expression changes of cyclin proteins, (**F**, **G**) P-MEK1/2 and P-ERK1/2, and (**H**) p53 and p21.

DU-145 cells were exposed to BA (0–1000 *μ*M) for 1, 2, or 7 days. No changes were apparent at 1 or 2 days, but at 7 days, the protein expression of cyclins A, B1, C, D1, E, and the phosphorylated form of MAPK signaler MEK (P-MEK1/2) decreased at 500 and 1000 *μ*M concentrations (Figure 2A–F). Phosphorylated ERK (P-ERK1/2) increased at intermediate exposures (100 and 250 *μ*M), relative to control, but was reduced by higher concentrations of BA (Figure 2B). The tumour suppressor gene p53 expression remained stable, but p21 decreased following 7-day exposures (Figure 2H).

### BA induces cytoskeletal alterations, while inhibiting cell attachment, migration, and invasion

**Figure 3:**
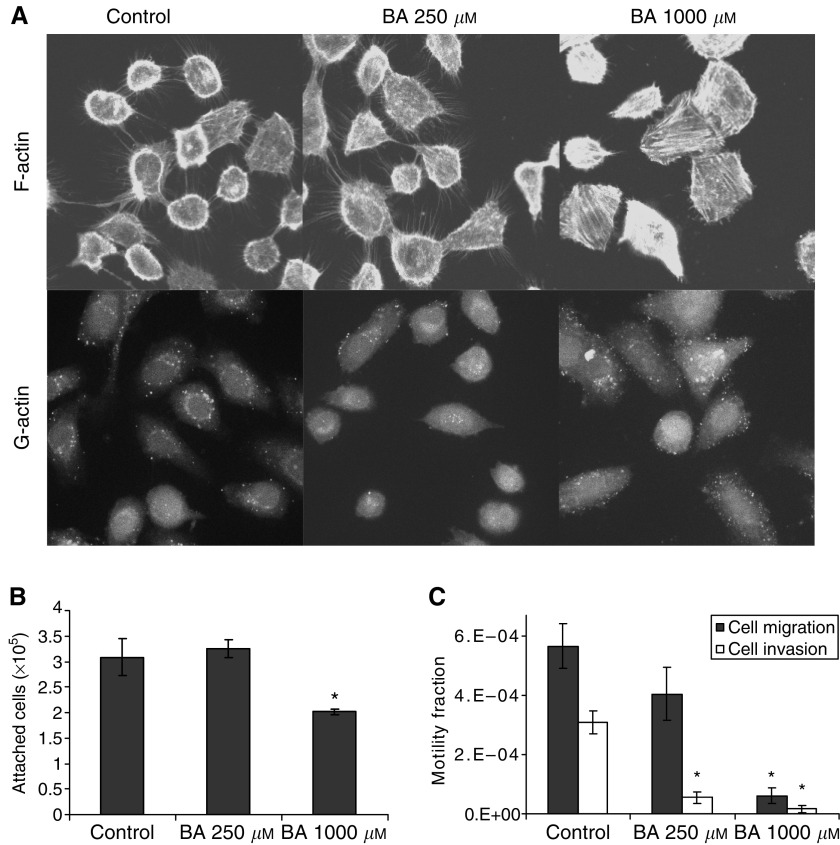
BA-induced changes in cell attachment, migration and invasion capacity, following 8-day exposure, in DU-145 cells. (**A**) Confocal images (63 ×) of BA-treated (0–1000 *μ*M) cells showing F-actin filopodia retraction, yet no effect on G- or F-actin relative expression. (**B**) Reductions occurred in attachment efficiency. ^*^ Statistically significant from control exposures (*P*-value <0.04). (**C**) Migratory and invasive capacity was reduced in treated cells; mean±s.e.m., *n*=3. ^*^ Statistically significant from control exposures (*P*-value <0.02).

Measurements were taken to assess cell attachment, migration, invasion, and intracellular cytoskeletal actin distribution to determine if BA (250 and 1000 *μ*M) exposure for 8 days had an effect on metastasis-related aspects of cancer cells. Staining for filamentous (F)-actin, a marker for intercellular connections and extensions such as filopodia, was decreased in cells exposed to high levels of BA. A total of 1000 *μ*M-treated cells had smooth-edges and were angular in appearance (Figure 3A). Intracellular globular (G)-actin expression was unaltered by BA exposure.

BA-treated cells show a reduction in attachment efficiency to polystyrene culture dishes, with a drop in plating efficiency of 34% at 1000 *μ*M (Figure 3B). With 10% FBS serving as a chemo-attractant, the capacity of DU-145 cells to migrate across an 8 *μ*m polycarbonate permeable membrane was reduced by 28 and 89%, by 250 and 1000 *μ*M BA, respectively (Figure 3C). The same trend was observed with invasion potential, where 250 and 1000 *μ*M BA pretreatments reduced matrigel invasion by 82 and 97% (Figure 3C).

### BA induces media acidosis and accumulation of acidic vesicles

**Figure 4:**
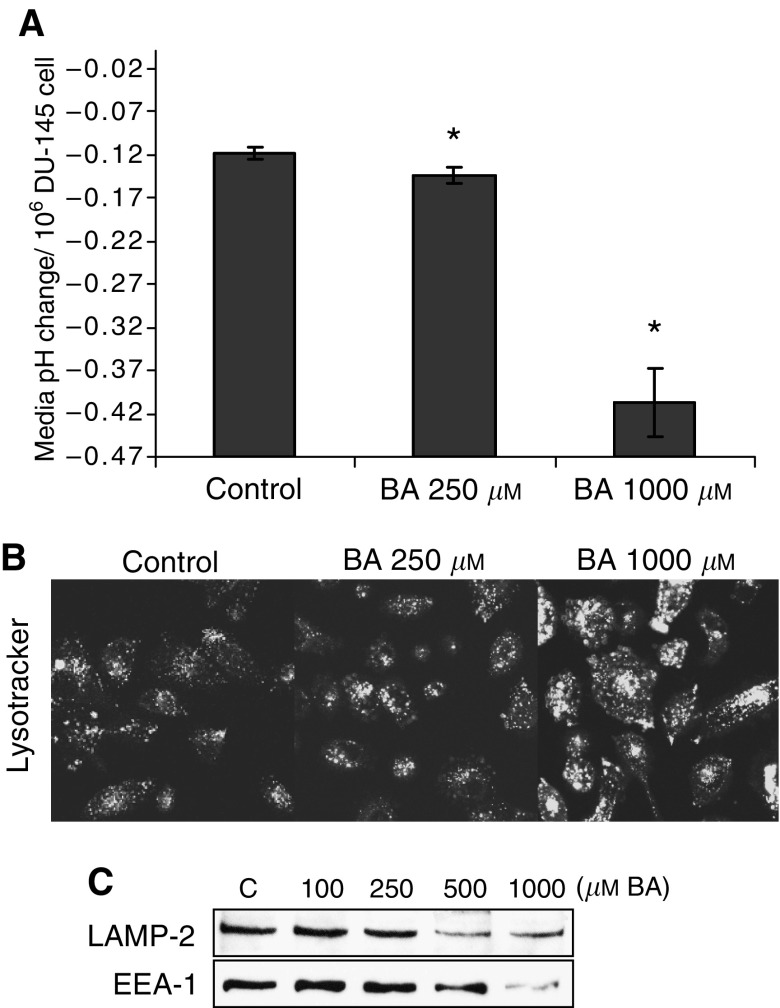
BA-induced media pH shifts and acidic vesicle accumulation, following 8-day exposure, in DU-145 cells. (**A**) pH shifts from 7.4 (per 10^6^ cells) recorded in BA-supplemented (0–1000 *μ*M) culture media, following overnight exposure, subsequent to 8-day treatment; mean±s.e.m., *n*=3. ^*^Statistically significant from control exposures (*P*-value <0.009). (**B**) Confocal microscopic images of BA-treated cells with Lysotracker (fluorescent marker of intracellular acidic compartments). (**C**) 7-day BA treatments (0–1000 *μ*M) led to downregulation of lysosome (LAMP-2) and the early endosome (EEA1) markers, as shown in Western blots.

Acidic yellowing of phenol red in culture media was more pronounced in BA treated cells. The pH of media was measured prior to (pH 7.4) and following exposure to DU-145 cells for 24 h, between the 7th and 8th days of culture. The pH for each concentration of BA (0, 250, and 1000 *μ*M) was then converted into an acidic shift from pH 7.4 per cell value. Chronically BA-exposed DU-145 cells acidified the surrounding culture media in a dose-dependent manner (Figure 4A). The number of acidic vesicles (measured using Lysotracker fluorescent probe) also increased in a dose-dependent manner, but both the lysosome-specific LAMP-2 protein and early endosome marker EEA1 decreased (Figure 4B and C). The concentrations of BA used in culture media displays no significant effect on media pH (data not shown). To exclude the possibility that BA might alter the buffering capacity of densely populated culture plates, cells were cultured to near-confluence in control media before exposure to BA-supplemented media (250 and 1000 *μ*M) for 24 h. The pH remained unchanged at all BA concentrations, showing acidity was not associated with the media, but instead with cell changes that occurred during the 7 day exposure (data not shown).

## DISCUSSION

Boron has a high affinity for oxygen and is present in aqueous solution, depending on pH, as either BA (B(OH)_3_) or borate (B(OH)_4_)^−^. Since the pK_a_ of the equilibrium between B(OH)_3_ and borate (B(OH)_4_)^−^ is 9.2, at intracellular pH (7.4) free boron exists as the weak Lewis acid, BA. BA, a small molecule with a mass of 61.83, is rapidly absorbed from the human intestine and excreted via urine with a half-life of 21 h (Jansen *et al*, 1984, Schou *et al*, 1984). There is no evidence supporting metabolism of BA in any animal species (EPA, 1991). BA does bind to molecules with *cis*-hydroxyl groups, as established through mass spectrometry and NMR analysis identifying a high affinity for the ribose moieties of NAD^+^, and a somewhat lower affinity for mononucleotides (Kim *et al*, 2003). Nucleotide phosphorylation and loss of charge greatly reduces substrate affinity for BA (Kim *et al*, 2004).

### Morphology

Flow cytometry analysis showed that BA caused a reduction in cell volume, yet under light microcopic investigation cells appeared to have a larger diameter. We believe the DU-145 cell line is responding to higher concentrations of BA by rearranging its cell shape into a flattened, low-volume state (Figure 3A). These structural alterations in shape and size are likely contributing to the inability of the cells to proliferate, since increased cell volume and a rounding up from the attached substrate are both critical events during mitotic division (Lang *et al*, 2000; Fujibuchi *et al*, 2005). The observation that morphological alterations did not appear following 1 and 2 day BA exposures, but did at 8 days, argues the changes reflect secondary response of long-term treatment with BA.

The relative intensity of fluorescent staining for G- and F-actin was found to be unchanged, regardless of BA concentration, indicating a steady-state actin pool ratio. Although actin concentrations in general appear unaltered, F-actin-stained filopodia extending about the periphery of the cells was reduced by 1000 *μ*M BA. With actin serving an important cytoskeletal factor in cell migration and invasion (Lambrechts *et al*, 2004), the observed F-actin retraction in BA-treated cells suggests a reduced capacity to perform either. This interpretation was reinforced by the analysis showing a dose-dependent inhibitory effect on motility and invasion capacity, along with incompetence for reattachment (Figure 3B and C). Together, these results suggest that BA reduces the metastatic potential of the DU-145 cell.

In BA-treated cells, granularity increased in proportion to exposure concentration, possibly due to the formation of intracellular vesicles (Figure 1A and B). The origin and content of these vesicles is unknown, since fluorescent probes for acidic compartments, tubulin, and intracellular calcium all failed to colocalise (data not shown).

### Proliferation

The mechanism underlying the antiproliferative activity of BA has not been elucidated. One of the intriguing properties of BA is its ability to inhibit proliferation without causing a shift in cell cycle stage distribution or cell death (Barranco and Eckhert, 2004). In the current study, BA decreased the expression of five major cyclin proteins, all presumably playing significant roles in cell cycle progression (Figure 2A). Furthermore, the ability of antiproliferative agents to inhibit the expression of these proteins is important, since cyclins A, B1, E, and D1 have been correlated with prostate cancer aggressiveness (Mukhopadhyay *et al*, 2002; Maddison *et al*, 2004; Tsao *et al*, 2004).

The DU-145 cell line has a mutant p53 protein incapable of signalling through p21, so it was nonetheless surprising to see p21 expression reduced by BA exposure (Figure 2B) (Lecane *et al*, 2003). The downregulation of p21 helps to explain why BA does not shift DU-145 cell populations into a G1 arrest (Barranco and Eckhert, 2004, Shukla and Gupta, 2004).

BA's effects on growth has been shown to be parabolic in embryonic trout and zebrafish with poor embryonic growth occurring at very low and high concentrations (U-shaped curve) (Rowe *et al*, 1998). BA's growth effects are cell-type dependent with maximum growth occurring in *Saccharomyces cerevisiae* cells at <0.8 *μ*M BA, whereas 500 *μ*M of BA maximised proliferation in HeLa cervical cancer cells (Bennett *et al*, 1999; Park *et al*, 2004). Furthermore, in HeLa cells BA (300 *μ*M) was shown to stimulate the MAPK pathway in a bell-shaped fashion, with an initial induction of P-MEK1/2 and P-ERK1/2, followed by a decline in expression of P-MEK1/2 over time. In the present study, BA reduced P-MEK1/2 expression in a dose-dependent manner, yet increased P-ERK1/2 moderately at 250 *μ*M (Figure 2F and G). By way of Ras/Raf signalling, the phosphorylated form of MEK phosphoylates ERK, which then translocates to the nucleus and activates transcription factors relevant in proliferative induction. Thus, by upregulation of this pathway's activity, it appears that DU-145 cells are attempting to counter the BA-induced growth inhibition (Giehl, 2005). Expression of MEK, ERK, and all cyclins were not altered following 1 and 2 day treatments suggesting, as observed with cell morphological changes, these were not the primary effect of BA.

### Senescence

DU-145 cells were evaluated for *β*-galactosidase activity, a marker of senescence or reversible cellular quiescence (Coates, 2002). When enzymatic activity is measured at pH 4.0, it is thought to indicate an increase in lysosomal enzyme concentration, whereas enhanced activity at pH 6.0 reflects an increased lysosomal mass (Kurz *et al*, 2000). In our study, BA treatment increased the activity of *β*-galactosidase in a dose-dependent manner at pH 4.0, yet no activity was apparent at pH 6.0. However, the dose-dependent increase recorded at pH 4.0 suggests the BA induces some ‘senescent-like’ characteristics.

### Accumulation of acidic intracellular vesicles

A peculiar manifestation of BA treatment was discovered when the media of chronically exposed DU-145 cells became increasingly acidic (Figure 4A). This effect was dose-dependent and not due to changes in the buffering capacity of the media or BA itself. The documented accumulation of acidic intracellular compartments is supportive of an affiliation with the media pH shift, by either contributing directly to the environmental acidification, or rather resulting from it, as seen in breast cancer cells (Glunde *et al*, 2003). Initially, we believed the upregulation of acidic vesicles reflected an increase in lysosome organelles, yet the LAMP-2 protein, expressed on lysosomal membranes in prostate tissue, decreased in expression (Figure 4C) (Furuta *et al*, 1999). It was also possible that the acidic vesicles were early endosomes, yet the protein expression of early endosome marker EEA-1 was likewise reduced (Eskelinen *et al*, 2003). Further studies are needed to determine if this response is unique to cancer cells or a universal response to BA (Gatenby and Gillies, 2004). Interestingly, metabolic acidosis has been reported in a case of fatal BA poisoning (Restuccio *et al*, 1992).

### Conclusion

The rationale for this study was based on the fact that BA is (i) a natural constitute of human blood, (ii) readily absorbed with plasma levels determined by dietary intake, and (iii) there is epidemiological, animal, and cell culture evidence supporting its antiproliferative capacity in prostate cancer. In this report, we show that pharmacologically-relevant BA treatment causes DU-145 prostate cancer cells to convert to highly granular, low-volume, flattened cells that have a marked reduction in their capacity to migrate, invade matrigel, and attach to synthetic substrates. Reduction in the expression of proliferation-relevant proteins, along with the upregulation of *β*-galactosidase activity, ultimately leads to a nonproliferating entity reminiscent of a senescent-like cell. Finally, the resulting cell accumulates intracellular acidic vesicles, while acidifying its extracellular environment.
